# Strong beaming of microwave surface waves with complementary split-ring-resonator arrays

**DOI:** 10.1038/s41598-018-30555-x

**Published:** 2018-08-14

**Authors:** Emily Young, Joseph A. Dockrey, Alastair P. Hibbins, J. Roy Sambles, Christopher R. Lawrence

**Affiliations:** 1 Department of Physics and Astronomy, University of Exeter, Stocker Road, Exeter, EX4 4QL UK; 2 QinetiQ, Cody Technology Park, Ively Road, Farnborough, GU14 0LX UK

## Abstract

A thin copper sheet, populated by an array of complementary split ring resonators, presents strong surface wave beaming in orthogonal directions at two distinct frequencies. This simple array is significantly thinner than existing single frequency beaming surfaces. The observed beaming frequencies are associated with the two lowest resonance modes of the split rings, and the beams are subwavelength in width and approximately non-diverging. The beaming is analysed through comparison of near-field scans of the surface-normal electric fields with numerical simulations.

## Introduction

Metal-dielectric interfaces are known to support surface plasmon polaritons (SPPs), electromagnetic (EM) fields coupled with longitudinal electron plasma density oscillations in the metal. These propagating surface modes are transverse magnetic (TM) in nature, with the electric fields decaying exponentially into both media^1^. Such well-bound surface states require that the frequency-dependent permittivity, *ε*, of the metal be predominantly real and negative^2^. For most Drude-like metals this occurs in the visible and near infra-red regime. By contrast at microwave frequencies for most metals *ε* becomes large and almost purely imaginary they approximate perfect electric conductors (PECs). As a result, surface modes are loosely bound to the interface. Such unbound states are referred to as Sommerfeld-Zenneck waves^3^.

However, as theorised in 2004^4^ and experimentally verified in 2005^5^, SPP-like behaviour can be mimicked at microwave frequencies through the introduction of subwavelength surface structure onto the metal in question. Such surface structuring effectively binds the surface modes to the interface, producing so-called ‘spoof’ or ‘designer’ SPPs. Many metasurfaces support such modes, from gratings and holes to more complex constructions such as Sievenpiper mushroom arrays^4,6,7^.

This present study centres on the phenomenon of beaming of such microwave surface waves. Beaming (or self-collimation) of SPPs appears to have been first presented in the optical regime in 1999^8^. Since then, there have been a number of papers on this topic, both in the optical regime^9–12^ and recently in the microwave range^13–15^.

Matthews^13^ explored an array of ceramic cylinders, fixed onto a polystyrene support, used to produce self-collimation for a frequency of 7.64 GHz. The resulting beam width is approximately five times the incident wavelength. An array of square metal holes fixed atop a metal backplate is studied by Kim *et al*.^14^. This supported beaming at a frequency of 15.60 GHz, with a beam-width of the order of twice the incident wavelength. Kim *et al*.^15^ use a square array of copper posts, fixed once more atop a metal backplate, displaying self-collimation of surface waves for a frequency of 6.06 GHz. In this last study, the team succeeded in achieving subwavelength beam width; however both papers present no analysis of the eigenmodes of the underlying structures. Thus far, all of the reported structures under investigation in the microwave regime have been notably bulky, on the order of tens of millimetres thick. They also require the use of a metallic backplates or polystyrene supports, and present beaming at a single frequency only.

In this present work, a similar self-collimation phenomenon is observed arising from the excitation of two different modes in a thin metal structure comprised of a planar array of complementary split ring resonator (CSRR) structures. These structures were first proposed in 2004^16^ as a way of achieving an effective negative *ε* in the microwave regime. A thorough characterisation of the microwave response of CSRRs was recently carried out by Navarro *et al*.^17^, comparing their resonance frequency, bandwidth and overall dimensions with well-known structures such as Sievenpiper mushrooms and gratings. The thin dimensions of the structures, their highly subwavelength unit cell size, and their broader bandwidth were listed as the key advantages of the CSRRs over the alternative structures.

By exploiting the advantages of CSRRs, this current study demonstrates that the self-collimation of surface waves, reported in previous work, may be achieved with structures of far more compact dimensions. By associating the beaming frequencies with the resonant eigenmodes of the underlying CSRR elements, a full explanation of the phenomenon is achieved. Significantly, the CSRRs display orthogonal beaming of surface waves at two completely separate frequencies, a phenomenon as yet unreported in the existing literature. Furthermore, both observed beams are highly subwavelength in width and approximately non-diverging in nature. Finally, the simplicity of the CSRR structures enables faster and easier fabrication than before. It is believed that these much thinner and simpler structures could prove useful for a number of engineering applications in the microwave regime, such as in surface based communications and device-to-device links where directionality matters and on devices where radar energy needs to be collected and channelled to reduce electromagnetic cross-section, such as wind turbine blades.

**Figure 1:**
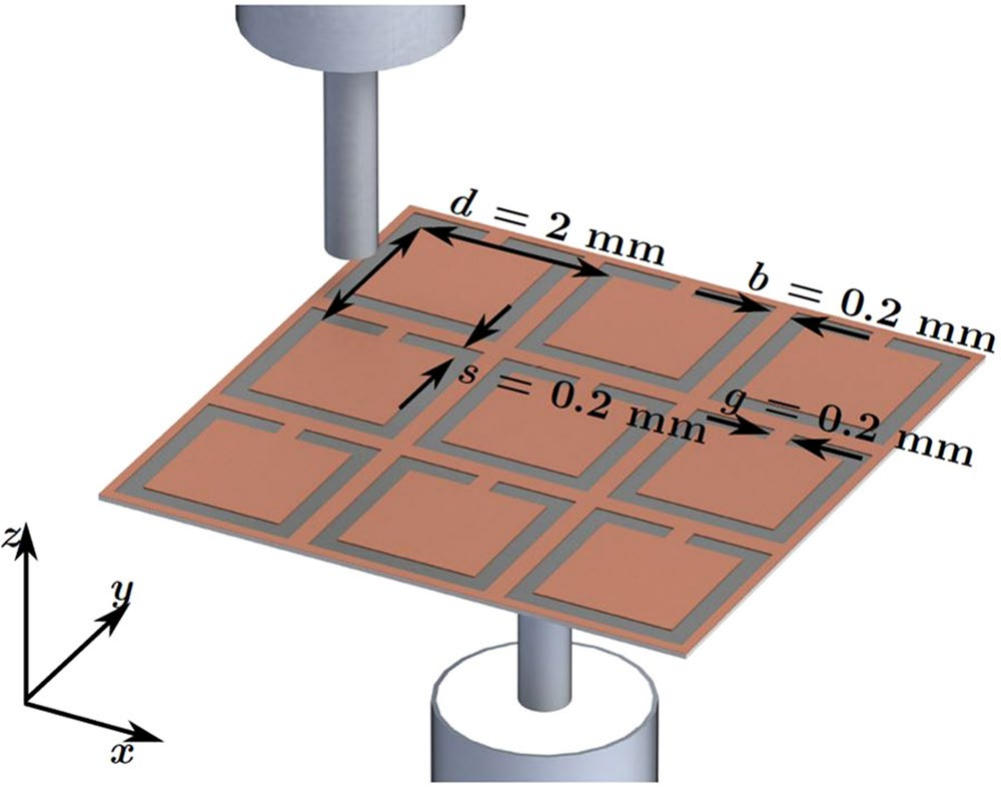
Schematic showing the experimental set up. An excitation probe illuminates the centre of a single CSRR cell from one side of the sample, and on the other side another probe scans across the complete surface. Total sample area is 200 by 280 mm.

Figure 1 shows the typical geometry of the CSRRs used in our experiments. The CSRRs are etched onto the copper sample in a square lattice of pitch *d* = 2 mm. Samples consist of 18 μm thick copper sheets backed by 50 μm thick polyethylene terephthalate (plastic) sheet.

The CSRRs were etched into the copper surface to expose the dielectric backing beneath.

Experimentally, stripped-end coaxial cables of outer diameter 2.1 mm and exposed wire diameter 0.5 mm, with exposed wire length of 1 mm, connected to a vector network analyser (VNA) were used as near-field probes to both excite and detect microwave surface waves on the sample surface. It is important to understand that our antenna probes are operating at frequencies below their resonance in order to minimise perturbation of the modes of interest. While we are not attempting to quantitatively measure the field strength of any particular field component, our probes will be most sensitive to the z-component of the electric field. In the discussion that follows, we make a comparison of the field distribution of the transverse-magnetic modes that propagate across the surface. The samples were placed in an *xyz* translation stage, with microwave absorber placed along all edges to minimize the effects of reflections. On one side of the surface, an excitation probe was used, being swept through frequency from 5 GHz to 70 GHz. On the other side of the sample, a similar probe was used to scan across the surface in the *xy* plane with step size of 0.25 mm in both directions measuring both the magnitude of the normal component of the electric field, *E*_*z*_, and the phase relative to an internal reference signal in the VNA. Both probes were arranged normal to the sample, at a distance of approximately 500 μm from the surface. A finite element method (FEM) model (COMSOL^18^), with the assumption of perfect conducting rings and lossless dielectric, was used to determine the eigenmodes of the system, and to model the expected resonant fields.

## Results

**Figure 2:**
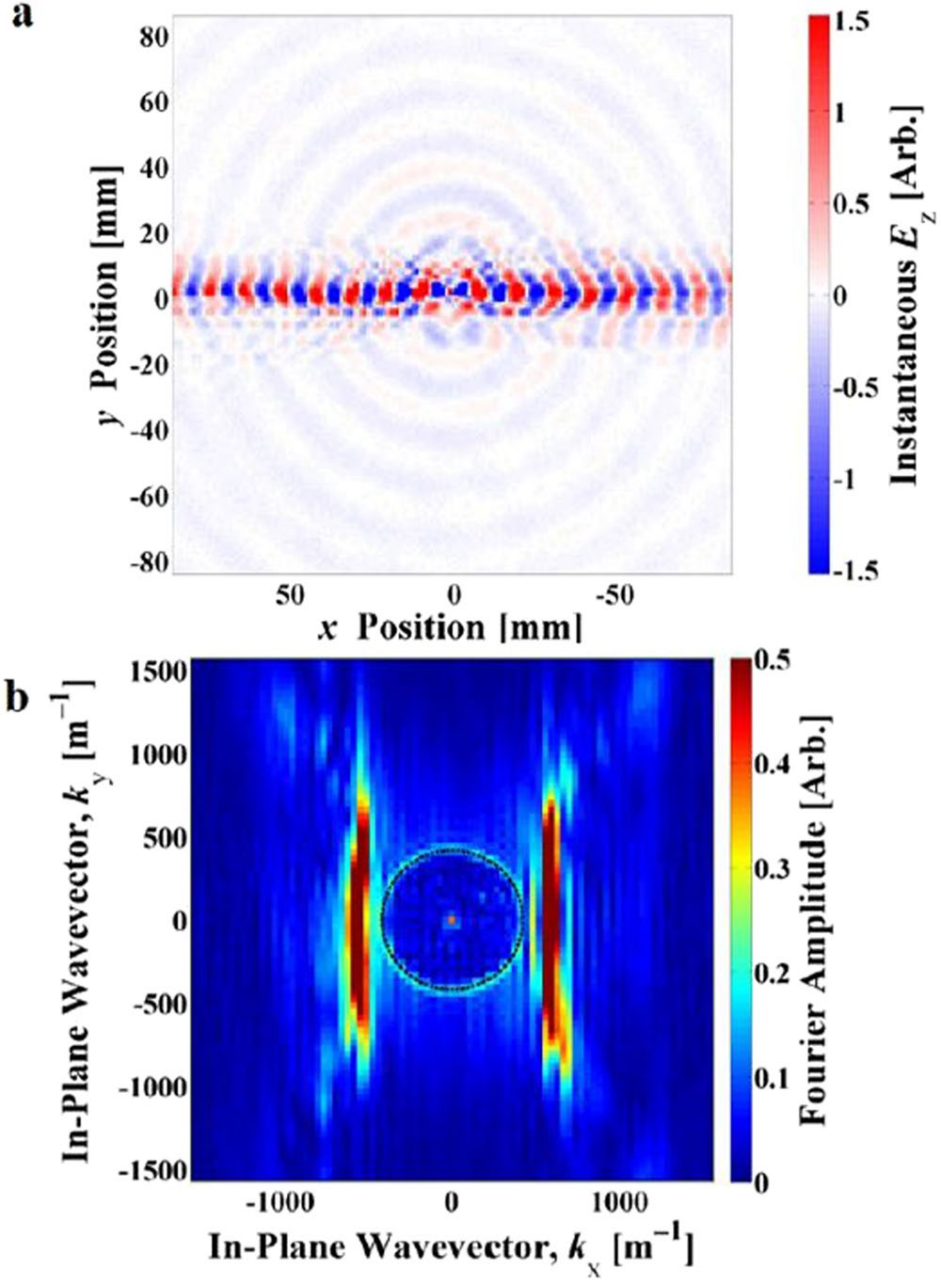
Experimental near-field scan results showing beaming at 20.00 GHz, with the excitation at the centre of the plot. (**a**) Real space plot of the normal component of the instantaneous electric field, *E*_*z*_; (**b**) Iso-frequency contour plot showing the Fourier amplitude of the in-plane wave vector components of the surface wave - flat contours indicate beaming. Colour scales are arbitrary.

**Figure 3:**
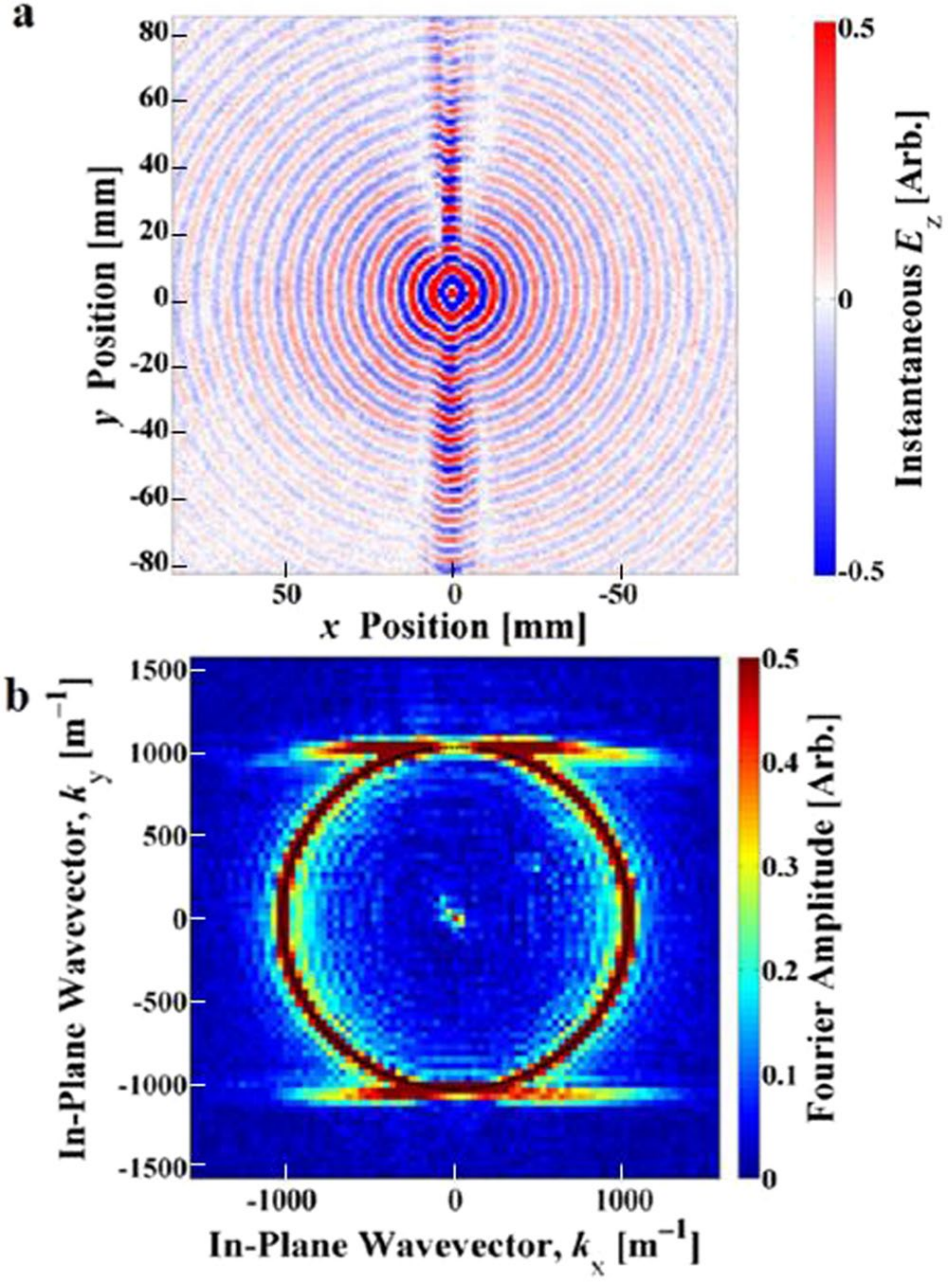
Experimental near-field scan results showing beaming at 49.15 GHz, with the excitation at the centre of the plot. (**a**) Real space plot of instantaneous *E*_*z*_; (**b**) Iso-frequency contour plot of the in-plane wave vector components of the surface wave. Colour scales are arbitrary.

Maps of the fields were obtained directly from the S12 measurements from the VNA at frequencies up to 70 GHz with a frequency step size of 0.05 GHz. Two frequency regions display strong surface wave beaming. The lower frequency beaming, shown in Fig. 2, occurs at close to 20 GHz and demonstrates self-collimation in a direction orthogonal to the CSRR split axis, along the *x*-axis of the sample. Note that this beaming is very tight with a width of order 8 mm compared to the wavelength of 15 mm. The higher frequency beaming, shown in Fig. 3, occurs around 49 GHz and here the surface waves are tightly collimated along the CSRR split axis, or the *y*-axis of the sample, with now a width of order the wavelength or about 2 unit cells.

A structured metal plate is known to support two families of surface modes, one with symmetric external magnetic fields parallel to the surface (*H*_//_) and one with antisymmetric external *H*_//_ fields. In the CSRR structures, we see the effects of the first and second order antisymmetric modes experimentally as the bright beams in the real-space plots of the measured time-averaged electric fields in Figs 2a and 3a. We also note the first order symmetric mode, which remains close to the light line, appearing as the non-collimated concentric background circles in Figs 2a and 3a.

By combining the measured time-averaged electric field magnitude and phase, we obtain the complex electric field. From this a 2D Fourier transform produces the *k*-space image of the excited eigenmodes of the system. This process has been used previously both in the optical regime (see supplementary information of^19^) and in the microwave regime^15^. Inspection of these *k*-space iso-frequency contours reveals information about the propagating modes supported in the system.

These iso-frequency contour maps, shown in Figs 2b and 3b, demonstrate the highly anisotropic response of the surface. Again we observe the different mode symmetries, with the first and second order antisymmetric mode contours elongating from circles into straight lines, which leads to the strong collimation of power or beaming. The symmetric mode remains largely close to the light line, appearing as the circle in the iso-frequency contours of Figs 2b and 3b.

Power propagation is given by the group velocity:

1 $${{\boldsymbol{v}}}_{{\boldsymbol{g}}}={\nabla }_{{\boldsymbol{k}}}\omega (k).$$

So for iso-frequency contour plots, the group velocity is normal to the k-vector contour lines^20^:

2 $${{\boldsymbol{v}}}_{{\boldsymbol{g}}}.d{\boldsymbol{k}}=0.$$

Bearing this in mind, it is clear that straight regions of the iso-frequency contour lines will set all normals for the straight region parallel to each other, forcing the power (group velocity) to travel in a straight and non-diverging path. Thus, the appearance of flat contour lines in the experimental iso-frequency data in Figs 2b and 3b accords fully with the beaming of the surface wave power.

**Figure 4:**
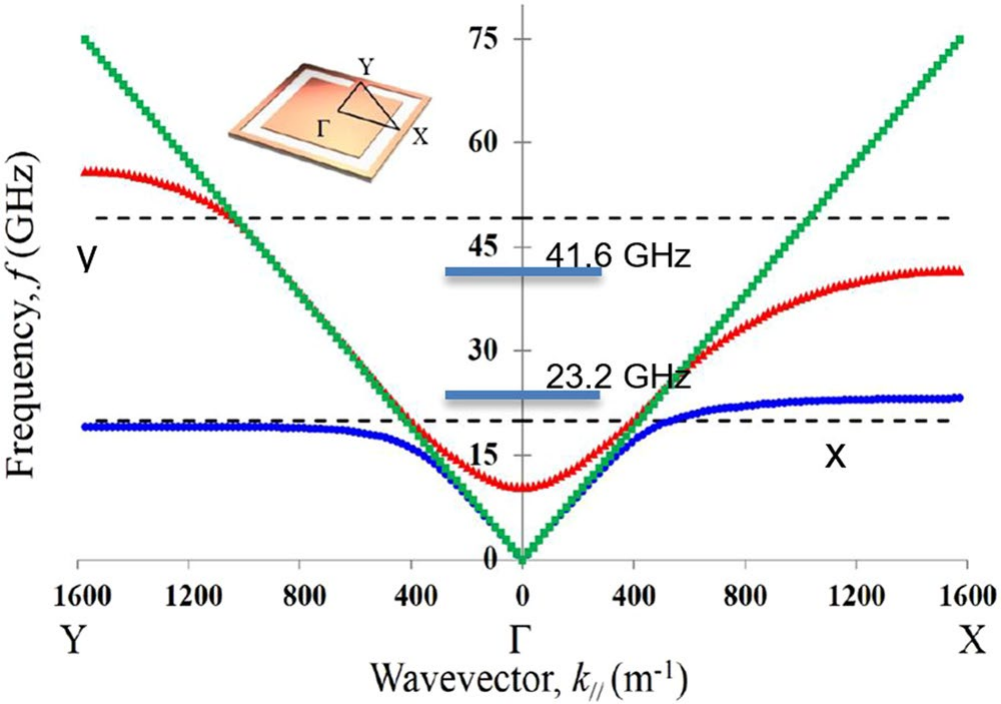
Model dispersion for the eigenmodes of the FEM model, plotted in the first Brillouin Zone. The lower mode in blue is the first order antisymmetric mode giving the 20.00 GHz x-direction beaming. The mode shown in red is the second order antisymmetric mode giving the 49.15 GHz y-direction beaming. The green points are the first order, isotropic, symmetric mode. Dashed lines show the positions of the lower (20.00 GHz, x-beaming) and higher (49.15 GHz, y-beaming) beaming frequencies. The lowest two mode resonant frequencies are indicated by the horizontal blue lines.

This strong power beaming anisotropy for this surface observed in experimental results is further verified by plotting model dispersion curves obtained from FEM^18^ simulations by solving for the eigenmodes of an infinite periodic array of these CSRRs, which are treated as perfect conductors. Results from such modelling is shown in Fig. 4. Here, the difference between the supported frequencies of waves travelling in the *Γ-X* direction compared to waves travelling in the *Γ-Y* direction is clear.

## Discussion

When attempting to describe this highly directional propagation of microwave surface wave power over the CSRR samples, it is helpful to understand the origin of the surface mode. ‘Spoof’ SPPs are a result of the collective excitation of resonances within the surface of a structured metal. The dispersion of the surface waves is dictated by the anticrossing of a grazing photon with a localised, in this case split ring, resonance of the system in question. Any periodic collection of resonant elements (often referred to as ‘meta atoms’) will produce such an effect. The branch of the dispersion curve beyond the light line (grazing photon) corresponds to the localised surface mode, ‘spoof’ surface plasmon. Whilst there are ‘spoof’ SPPs associated with many higher order resonances associated with the CSRRs, the beaming observed in the surface-normal electric field data shown in Figs 2 and 3 arises from just the first and second order resonances. Consequently in the following simulated results fields related to these two modes only are shown.

**Figure 5:**
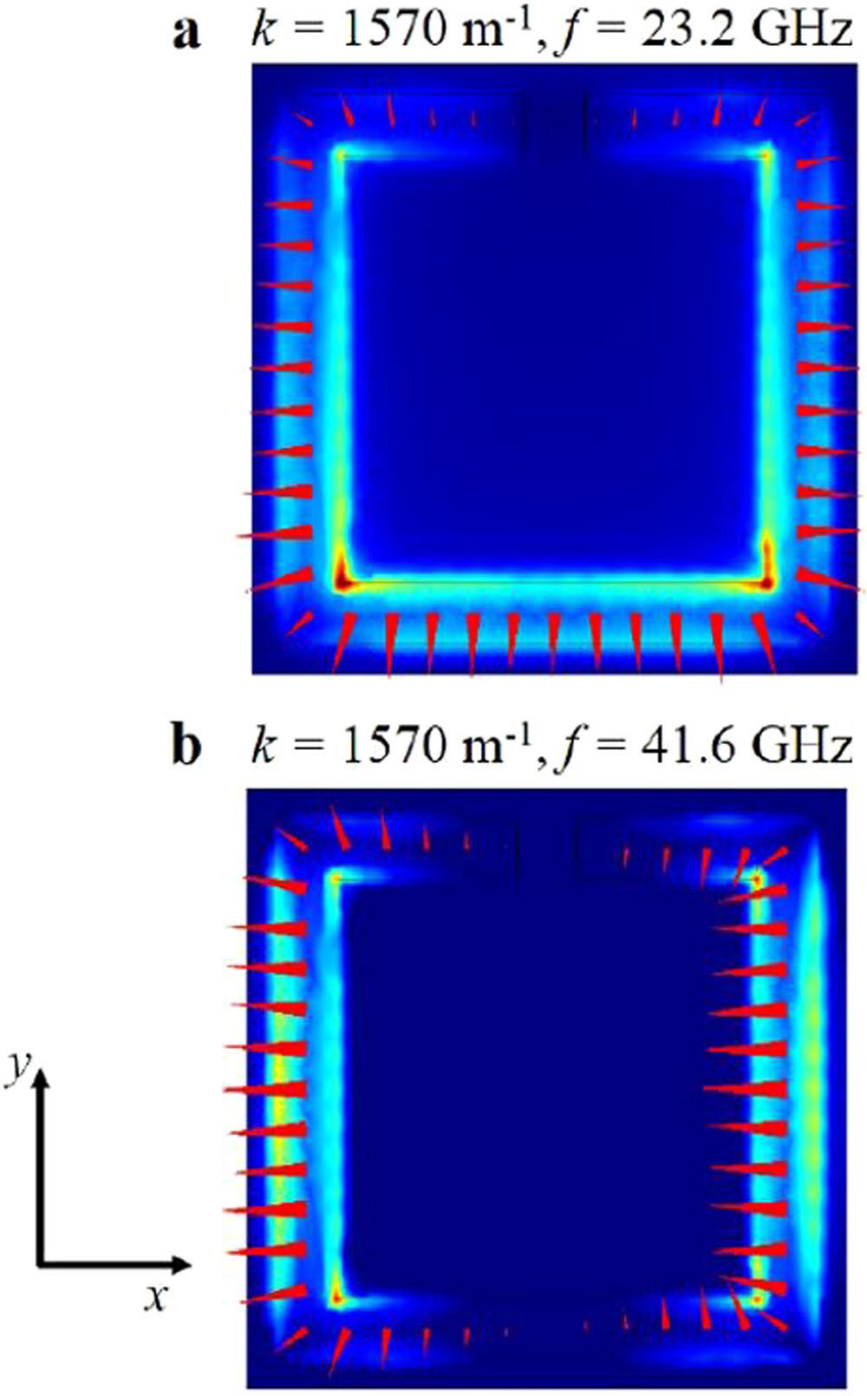
Simulated eigenmodes of the CSRR showing the electric field magnitude (background colour - red indicates high field and blue indicates low field) and direction (red vector arrows) plotted at the Brillouin Zone edge for (**a**) the lower and (**b**) the higher frequency modes.

Figure 5 shows the electric fields calculated by the FEM model for the eigenmodes of the CSSR. The lower frequency eigenmode is characterised by a *π* phase change in the electric field around the perimeter of the split ring, with a zero in electric field intensity at the split. This is the fundamental (half-wavelength) mode of the element. By contrast the higher mode fits a full wavelength around the ring and has two field zeros, with one sitting at the centre of the ring length. The length of the conductor is about 6.2 mm, which would naively give a fundamental resonance of 24.2 GHz with the harmonic at 48.4 GHz. Both simple estimates are close to the computed values of 23.2 GHz and 41.6 GHz.

The fields along the left and right sides of the CSRR for the lower mode of the model in Fig. 5a are equal and opposite, leaving one remaining field component along the *y*-axis of the ring. Taken in isolation, one can compare this component to the fields that would occupy a rectangular hole of the same length along this lower side, oriented along the *x*-axis. Similarly, the two high field regions of the higher mode in Fig. 5b could be compared to two rectangular holes oriented along the *y*-axis. The dispersion surfaces arising from arrays of rectangular holes have already been shown to be distorted^21^, with elongation of the iso-frequency contours occurring along the short axis of the rectangles. Thus, by representing the CSRR structure in this way, the switch in orientation of surface wave beaming between the two modes may be explained.

To test this analogy, a further sample was created with an extra split introduced into the lower ring section where the main field component for the lower frequency mode resides. The addition of this new split was designed to remove this high field region, thus effectively removing the part of the ring acting as the single rectangular hole for the lower mode and hence destroying the beaming previously seen along the *x*-axis.

**Figure 6:**
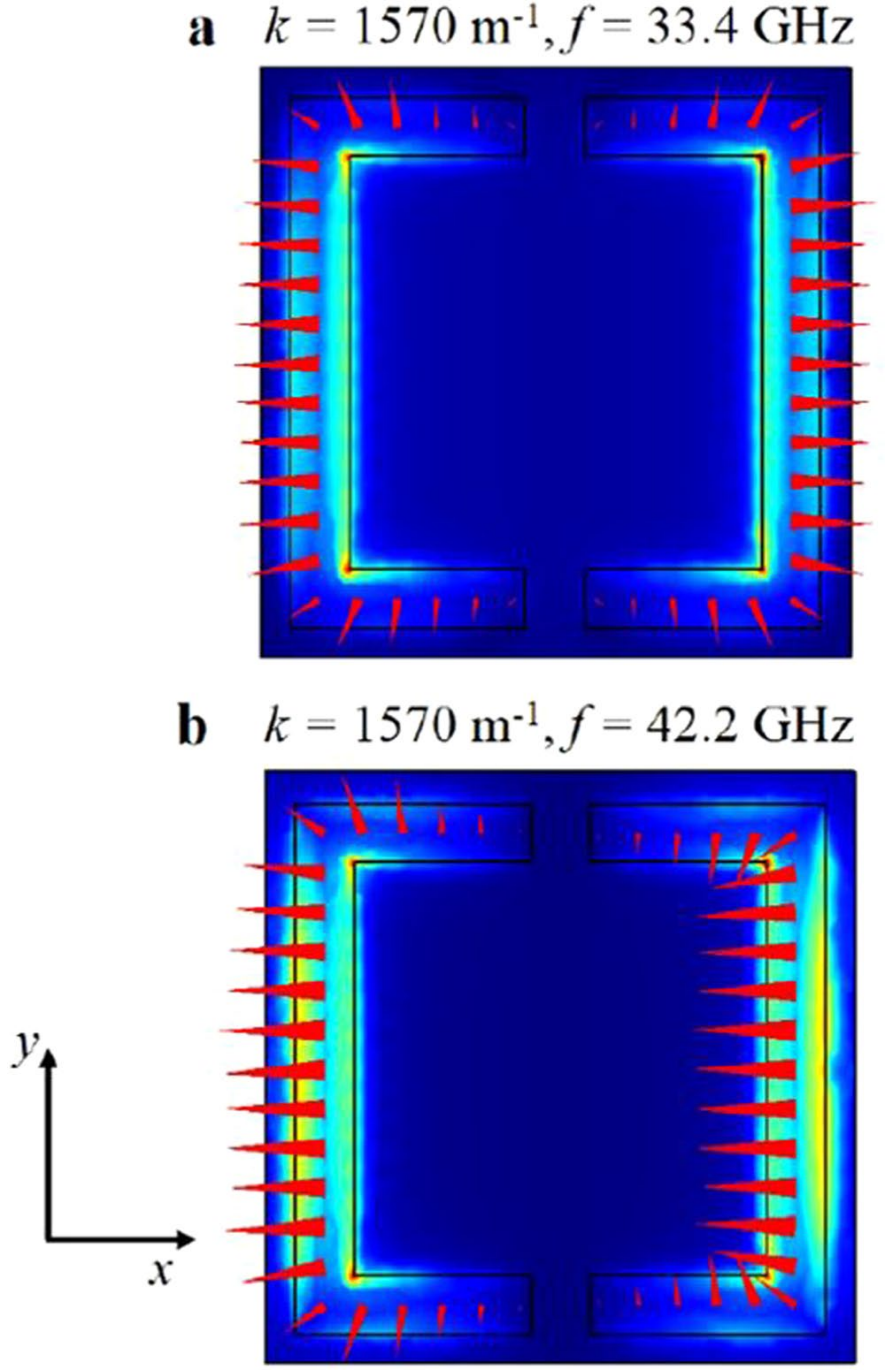
Simulated eigenmodes of the double split CSRR showing the electric field magnitude (background colour - red indicates high field and blue indicates low field) and direction (red vector arrows) plotted at the Brillouin Zone edge for (**a**) the lower and (**b**) the higher frequency modes. Note that although the model still shows a solution for the lower mode (shifted up in frequency), there is no longer an overall component of the electric field. The higher mode is largely unaffected by the addition of the extra split.

First, a simulation of this sample was carried out to verify the effect of the extra split on the supported eigenmodes of the structure. This is shown in Fig. 6. The lower mode still exists as an eigenmode of the structure; however it is significantly changed in frequency and the fields now display only the equal and opposite components on the left and right sides of the ring. The component contributing to the lower mode and its associated beaming has been removed. As expected, the higher order mode remains.

**Figure 7:**
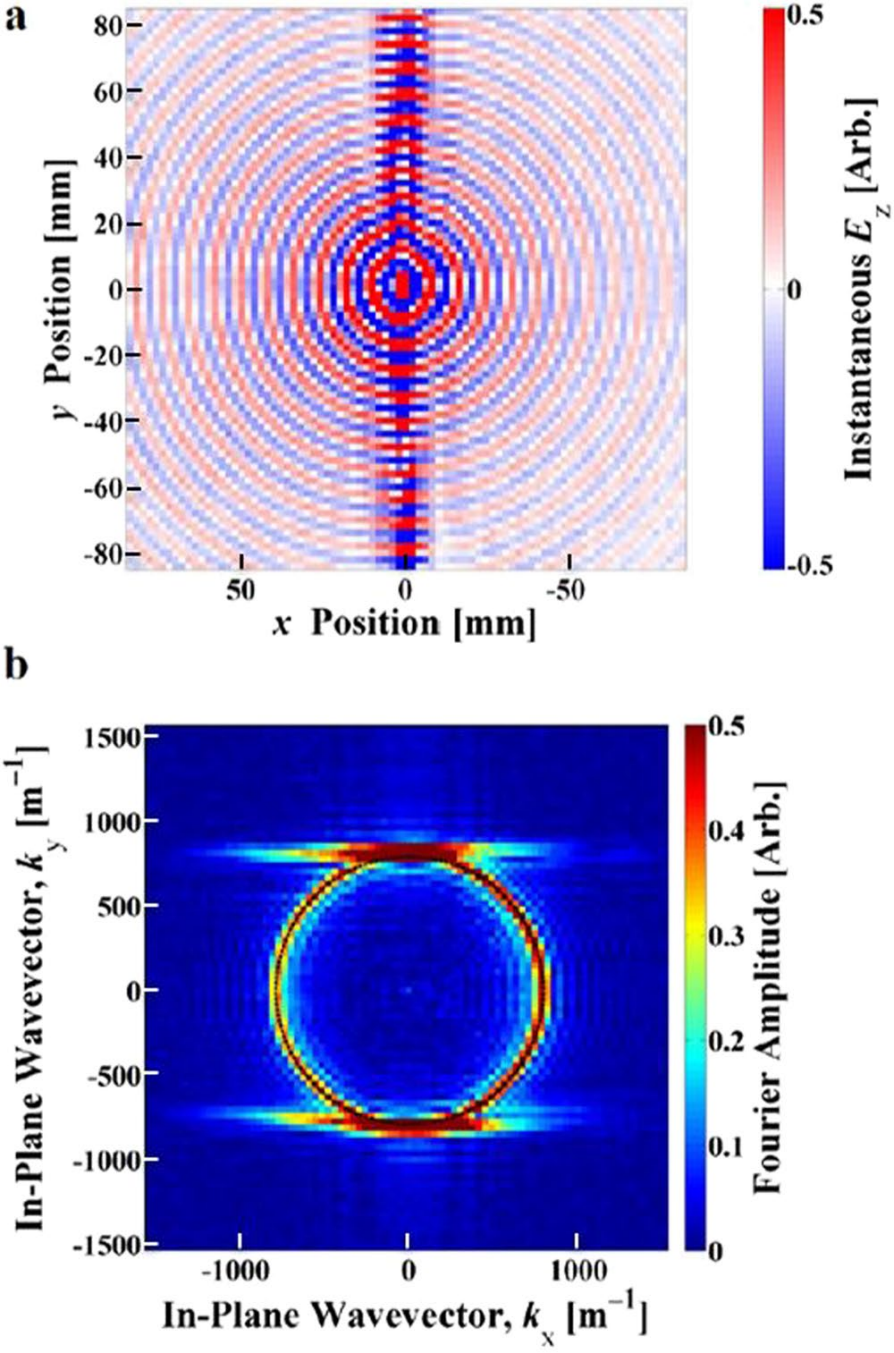
Experimental near-field scan results showing beaming at 37.75 GHz for the double split CSRR array with excitation as the centre. (**a**) Real space plot of the normal component of the instantaneous electric field, *E*_*z*_; (**b**) Iso-frequency contour plot of the in-plane wave vector components of the surface wave.

The doubly split sample was then fabricated and scanned in the same way as the original CSRRs. The fields from the exciting antenna no longer couple to the lower mode responsible for beaming along the *x*-axis while the remaining mode displays beaming along the *y*-axis (see Fig. 7), similar to the 49.15 GHz mode of the original CSRR structure. The removal of the lower mode supports the analogy presented above, and explains why the singly split CSRRs display perpendicularly oriented beaming of the surface wave power for the two modes.

The beaming frequency of the remaining mode for the doubly split CSRR has, however, been reduced to 38 GHz somewhat below the calculated resonant frequency of 42.2 GHz as compared to the original beaming at 49.15 GHz, significantly above the original resonant frequency of 41.6 GHz. This change arises from the strength of diffractive scattering and the resulting band-gap at the edge of the first Brillouin zone.

In summary, complementary split ring resonator arrays display propagating surface modes, and for the lower two of these the power in the surface wave is at selected frequencies very strongly collimated, being confined to a width somewhat less than the wavelength. This behaviour can be explained through analogy to rectangular holes, which display similarly anisotropic dispersion. In contrast to previous work demonstrating surface wave beaming, the planar CSRR array presents a far thinner structure capable of supporting this phenomenon, and the simplicity of these thin structures allows for easier sample fabrication. Additionally, the arrays also display beaming at two separate frequencies, in orthogonal directions. Such structures could therefore prove useful for engineering applications in the microwave frequency regime.

## Data Availability

All data created during this research are openly available from the University of Exeter’s institutional repository at https://ore.exeter.ac.uk/.
